# A family pedigree of malignancies associated with *BRCA1* pathogenic variants: a reflection of the state of art in China

**DOI:** 10.1186/s13053-019-0126-4

**Published:** 2019-09-10

**Authors:** Wenhui Li, Lei Li, Ming Wu

**Affiliations:** Department of Obstetrics and Gynecology, Peking Union Medical College Hospital, Chinese Academy of Medical Sciences, Shuaifuyuan No 1, Dongcheng District, Beijing, 100730 China

**Keywords:** *BRCA* mutations, Ovarian cancer, Genetic counseling, Risk-reducing salpingo-oophorectomy

## Abstract

**Background:**

Little is known about the status of genetic counseling for ovarian cancer in China.

**Case presentation:**

We report a four-generation Chinese family with several types of cancer. The proband was a patient with high-grade serous ovarian cancer (HGSOC) who was found to harbor a pathogenic *BRCA1* variant. Cosegregation analysis identified 7 of 9 relatives with the same deleterious variant. One month after the genetic test, one female carrier 54 years of age was diagnosed with stage IVB HGSOC, and another female 55 years of age accepted risk-reducing salpingo-oophorectomy, which revealed occult cancer of the fallopian tube (Stage IA).

**Conclusions:**

Genetic counseling and testing for ovarian cancer in China have fallen behind international trends. Innovative studies and practices are urgently needed to establish models for cancer screening, prevention and treatment.

## Background

Ovarian cancer is the leading cause of cancer-related mortality among females without an effective screening strategy [1]. Inherited breast cancer susceptibility gene 1/2 (*BRCA1/2*) mutations, the most commonly mutated genes in high-grade serous ovarian carcinoma (HGSOC), account for the majority of cases of familial ovarian cancer [2]. As many as 13 to 20% of patients diagnosed with epithelial ovarian cancer (EOC) carry a *BRCA1/2* mutation [3], and the lifelong cumulative risk of EOC is reportedly 44 and 17% for *BRCA1* and *BRCA2* mutation carriers, respectively [3]. Genetic counseling and testing are recommended for women with a history of ovarian cancer, and further cosegregation analysis is essential for the blood relatives of patients with positive test results. Those who harbor *BRCA1/2* deleterious mutations should be offered intensive screening and prevention strategies, and risk-reducing salpingo-oophorectomy (RRSO) should be recommended at an appropriate age for *BRCA* mutation carriers [4, 5]. However, in China, there are few official genetic counseling clinics and/or models for EOC.

We report a pathogenic *BRCA* variant-related family pedigree of malignancies. We also searched PubMed, EMBASE and SCOPUS, databases of clinical trials (*clinicaltrial.gov*, and *who.int/ictrp/network/en/*)*,* and the website of the Chinese Human Genetic Resources Management Office of the National Ministry of Science and Technology (*http://www.most.gov.cn/bszn/new/rlyc/jgcx/index.htm*) for reports and/or studies on the topics of genetic counseling of gynecologic cancer in China. We include a discussion on the limitations, potential strategies, and policies regarding genetic counseling for the Chinese population.

## Case presentation

**Fig. 1:**
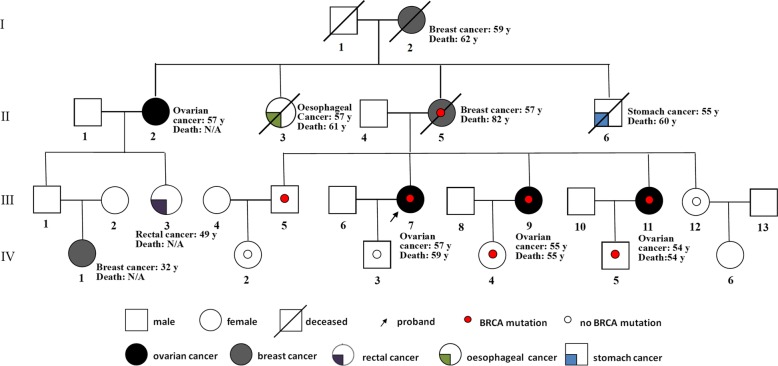
Pedigree structure of the Chinese family with hereditary ovarian syndrome. The affected family members are indicated with shading. Squares and circles denote males and females, respectively. Roman numerals indicate generations. The arrow indicates the proband (IV-7). A small red circle indicates which family members were tested for mutations and found to carry the mutation. A small hollow circle indicates which family members were tested and found not to carry the mutation. The numbers after cancer sites indicate the age at diagnosis. The age of death is reported if known. D, deceased

This study was approved by the Institutional Review Board of Peking Union Medical College Hospital. All involved family members provided informed consent in anticipation of the study. Data for all family members, including sex, tumor type, and age at death, were collected in detail (Fig. 1, according to the recommendations of the National Society of Genetic Counselors [6]). The proband (III-7) was a 59-year-old female diagnosed with HGSOC at the age of 57 years. Her grandmother (I-2) and mother (II-5) both died of breast cancer. The proband’s mother had two sisters, one of whom died of esophageal cancer at the age of approximately 60 years (II-3). The other sister had ovarian cancer of unknown type (II-2), and she had a daughter diagnosed with rectal cancer, an unaffected son and a granddaughter with breast cancer. The proband’s uncle (II-6) died of stomach cancer in his 60s. Before January 2018, four siblings of the proband did not show any evidence of malignancy.

In January 2018, the proband and her family members were referred to the genetic counseling clinics for gynecologic oncology at Peking Union Medical College Hospital. Germline sequencing using a 25-gene panel, i.e., *BRCA1*, *BRCA2*, *CHEK2*, *PALB2*, *BRIP1*, *EPCAM*, *TP53*, *PTEN*, *STK11*, *CDH1*, *ATM*, *BARD1*, *MLH1*, *MRE11A*, *MSH2*, *MSH6*, *MUTYH*, *NBN*, *NF1*, *PMS1*, *PMS2*, *RAD50*, *RAD51C*, *RAD51D* and *SMARCA4*, by BGI Clinical Diagnostic Laboratory (Shenzhen, China) was provided for the proband. Target-region capture and second-generation high-throughput sequencing were employed to analyze the exons and adjacent ±10-bp intron variations of relevant genes. The test revealed a heterozygous pathogenic deletion mutation in *BRCA1* exon 8, NG_005905.2 (NM_007294.3):g.25529_31240del, which was validated by quantitative polymerase chain reaction (qPCR) instead of the Sanger method. The qPCR results by BGI was used to validate the copy number variant (CNV) detected in our study. Specific primers were designed to evaluate exon CNVs. DNA samples were diluted to 25 ng/μl and added to a house mixture; qPCR was performed using an ABI StepOne real-time PCR system (BGI Clinical Diagnostic Laboratory, Shenzhen, China). The detailed testing information is provided in Additional file 1: Table S1. The variants were classified into 5 categories according to American College of Medical Genetics (ACMG) recommendations [7].

**Fig. 2:**
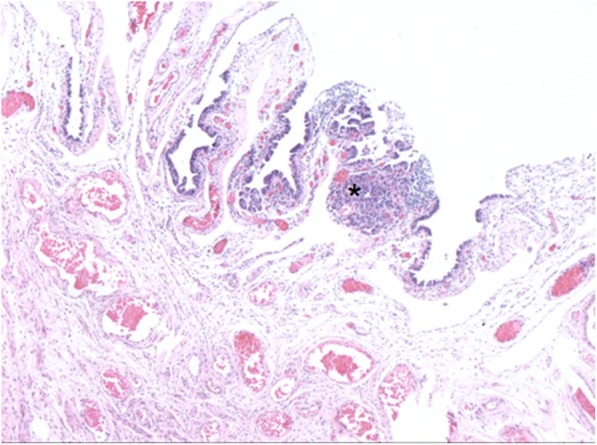
Hematoxylin and eosin stain of an infiltrating lesion of 0.5 mm (marked as asterisk) in the fallopian tube of III-11 after risk-reducing salpingo-oophorectomy

Thereafter, from February to March 2018, qPCR cosegregation analysis of the *BRCA1* mutation was conducted for nine family members (II-5, III-5, − 9, − 11, − 12, IV-2, − 3, − 4, − 5). The results are presented in Fig. 1. Despite thorough clarification and education about the potential negative influence of testing on juveniles, one 14-year-old girl accepted cosegregation analysis at her parents’ request and consent after the permission from the Institutional Review Board of the study center. Counseling about cancer prevention and targeted therapy was provided to all adult mutation carriers. Due to their age, a suggestion of RRSO was provided to both sisters of the proband (III-9 and III-11; 54 and 55 years, respectively). In April 2018, just 1 month after the genetic test, one of the proband’s sister (III-11) developed massive ascites and metastasis to the supraclavicular lymph nodes. Pathology of the biopsied material confirmed stage IVB HGSOC. She accepted neoadjuvant chemotherapy and interval debulking surgery. In May 2018, the other sister of the proband (III-9) accepted RRSO with hysterectomy. Pathological examination in accordance with the Sectioning and Extensively Examining the Fimbriated End Protocol (SEE-FIM) [8] revealed an infiltrating lesion of 0.5 mm in the right fallopian tube (Fig. 2), which coincided with the diagnosis of fallopian tube carcinoma at International Federation of Gynecology and Obstetrics (FIGO) stage IA. After sufficient discussion, she refused further staging surgery or prophylactic chemotherapy and decided to follow meticulous supervision mainly consisting of CA125 and transvaginal ultrasound. She remained well after the diagnosis at 7 months of follow-up. The chronology of the genetic tests and RRSO for the entire family is listed in Table 1.

**Table 1:** Chronicle of the genetic tests and risk-reducing salpingo-oophorectomy (RRSO) for the reported family

Date	Events
January, 2018	The proband (III-7) received a 25-gene panel targeted genetic test and was confirmed as deleterious *BRCA1* carrier.
February to March, 2018	Pedigree validation was performed for nine family members (II-5, III-5, 9, 11, 12, IV-2, 3, 4, 5), and seven (II-5, III-5, 9, 11, 12, IV-4, 5) were confirmed as deleterious mutation carriers.
April, 2018	One family member (III-11) was diagnosed with high-grade serous ovarian carcinoma of stage IV. She accepted neoadjuvant chemotherapy and interval debulking surgery.
May, 2018	One family member (III-9) accepted RRSO and hysterectomy, and was diagnosed with primary carcinoma of the fallopian tube of Stage IA (lesion size of 0.5 mm). She asked for observation and refused further therapy.

## Discussion

The case presented herein is a classical hereditary malignancy caused by *BRCA* pathogenic variants. Nevertheless, in China, both genetic counseling/testing and prophylactic intervention have fallen behind the process required based on international guidelines, which is a reflection of the current status. Chinese oncologists and patients are both seriously uninformed regarding tests for inherited ovarian cancers because of poor education and counseling [9, 10], despite several national cohort studies about *BRCA* germline mutations [11–13]. In our search of genetic counseling for gynecologic cancer in China, no specific studies were retrieved, except for two reports from Hong Kong [14, 15] and two ongoing trials (NCT03015376 and NCT03291106, *clinicaltrials.gov*). To our knowledge, there is only one genetic counseling clinic on gynecologic malignancy operating in mainland China [9], and no counselors have received formal training, assessment or certification. Thus far, the Chinese medical care system has not covered the expenses of mutation screening for high-risk individuals and/or families, even though cost concerns may contribute to low testing rates of population-based samples for at-risk cancer survivors [16]. All of these deficiencies have limited the development and promotion of genetic counseling for hereditary ovarian cancer and Lynch syndrome-associated endometrial cancer.

Timely and precise counseling, testing and cosegregation analysis should be offered to all women recently diagnosed with epithelial ovarian cancer as well as to their relatives [17], which is generally welcome but is contextualized within the broader experiences of these women [18]. Nonetheless, genetic testing was provided to most of the females of older ages in our report. The lack of testing and intervention resulted in the unfortunate event of rapidly evolved EOD in the proband’s sister (III-11). However, genetic testing was provided for a 14-year-old juvenile at her parents’ insistence, a step that is against the current general recommendation [4], as malignancies associated with *BRCA1/2* mutations generally have an adult onset. Decisions about whether to offer genetic testing and screening should be driven by the best interest of the child [19]. Despite extensive counseling about the possible negative effect of testing, the girl’s parents insisted on the test. However, there are no existing rules, regulations, or laws regarding appropriate genetic testing in China. In general, the potential impairment and injury of genetic testing on juveniles need further exploration. Rational counseling and informed consent should follow evidenced-based guidelines and the specific cultural environment and should be driven by the best interest of the child [19]. Regardless, genetic testing for asymptomatic children in this report is beyond the authors’ consensus and general practice, and the flaw and limitation in current professional and administrative regulations should be addressed in a timely manner.

An approximate fourfold increase in precancerous lesions or occult cancer was reported to result from the use of the SEE-FIM protocol as opposed to the classical method [20]. Within the context of the traditional culture and physician-patient relationship, prophylactic interventions have been more difficult to perform than general testing, and it was reported that Chinese *BRCA* mutation carriers have higher rates of surveillance than prophylactic surgery or the use of chemoprevention drugs [9]. To date, there is only one registered clinical trial regarding RRSO with the SEE-FIM protocol in China (NCT03294343, *clinicaltrials.gov*). The siblings of the proband in our study were at relatively older ages when they received genetic testing and RRSO, likely leading to the occurrence of advanced HGSOC (III-11, 54 years old) and stage IA fallopian tube carcinoma (III-9, 55 years old).

A national policy is critical for counseling, testing and prophylactic surgery for populations at high risk of cancer [21], especially in China, a developing country with a vast population. Improvements in healthcare infrastructure will be required to realize population-level benefits from *BRCA* genetic counseling and testing [22]. Furthermore, resources available within national and local agencies, professional societies, and in advocacy and community groups are critical for the successful implementation of cascade testing [23]. A public health approach for low-income women can be successful when integrated with the efforts of existing safety net organizations [24]. Possible approaches to overcome the disparity of genetic counseling include the use of patient navigators, online social media, or electronic medical records-based decision support aids [25]. In the future, the integration of genetic testing for *BRCA1/2* into the social health insurance for women with EOC should be developed as a policy of national public health care for Chinese patients.

## Conclusions

Very few clinical trials and counseling clinics on hereditary ovarian cancer are available for the majority of patients and their families in China. Appropriate and timely cosegregation analysis and interventions for reducing ovarian cancer risks are urgently needed, as is a national policy supporting and regulating such research and practices.

## Supplementary information

Additional file 1: **Table S1.** Details of the genetic testing for the proband. (DOCX 16 kb)

## Data Availability

All data generated or analyzed during this study are included in this published article and its supplementary information files.

## Abbreviations

HGSOC: High-grade serous ovarian carcinoma
QPCR: Quantitative polymerase chain reaction
RRSO: Risk-reducing salpingo-oophorectomy
SEE-FIM: Sectioning and Extensively Examining the Fimbriated End Protocol

## Supplementary material

**Supplementary information** accompanies this paper 10.1186/s13053-019-0126-4.
