# Supplementary material for: A family pedigree of malignancies associated with BRCA1 pathogenic variants: a reflection of the state of art in China
Source: Hered Cancer Clin Pract. 2019 Sep 10;17:26. doi: 10.1186/s13053-019-0126-4 (PMC6734459; doi:10.1186/s13053-019-0126-4)
Supplement: Supplementary file 1 — Table S1. Details of the genetic testing for the proband. (DOCX 16 kb) [file 13053_2019_126_MOESM1_ESM.docx]

Table S1

Variation identified at coding exons and exon-intron (+/-10 base pairs) boundaries in the proband*.

| No. | Gene | Transcript | Nucleotide change | Amino acid change | Gene locus | Heterozygosity | Rs No. | Functional change | Mutational type |
| --- | --- | --- | --- | --- | --- | --- | --- | --- | --- |
| 1 | *ATM* | NM_000051 | c.3285-10delT | - | Intron22 | Het | rs1799757 | Splice | Benign |
| 2 | *ATM* | NM_000051 | c.5557G>A | p.Asp1853Asn | CDS36 | Het | rs1801516 | Missense | Benign |
| 3 | *BARD1* | NM_000465 | c.1519G>A | p.Val507Met | CDS6 | Het | rs2070094 | Missense | Benign |
| 4 | *BARD1* | NM_000465 | c.1134G>C | p.Arg378Ser | CDS4 | Het | rs2229571 | Missense | Benign |
| 5 | *BARD1* | NM_000465 | c.70C>T | p.Pro24Ser | CDS1 | Het | rs1048108 | Missense | Benign |
| 6 | *BRCA1* | NM_007294 | c.4837A>G | p.Ser1613Gly | CDS14 | Het | rs1799966 | Missense | Benign |
| 7 | *BRCA1* | NM_007294 | c.3548A>G | p.Lys1183Arg | CDS9 | Het | rs16942 | Missense | Benign |
| 8 | *BRCA1* | NM_007294 | c.3113A>G | p.Glu1038Gly | CDS9 | Het | rs16941 | Missense | Benign |
| 9 | *BRCA1* | NM_007294 | c.2612C>T | p.Pro871Leu | CDS9 | Het | rs799917 | Missense | Benign |
| 10 | *BRCA2* | NM_000059 | c.1114A>C | p.Asn372His | CDS9 | Het | rs144848 | Missense | Benign |
| 11 | *BRCA2* | NM_000059 | c.10234A>G | p.Ile3412Val | CDS26 | Het | rs1801426 | Missense | Benign |
| 12 | *BRIP1* | NM_032043 | c.2755T>C | p.Ser919Pro | CDS18 | Hom | rs4986764 | Missense | Benign |
| 13 | *CDH1* | NM_004360 | c.48+6C>T | - | Intron1 | Hom | rs3743674 | Splice | Benign |
| 14 | *MSH2* | NM_000251 | c.211+9C>G | - | Intron1 | Hom | rs2303426 | Splice | Benign |
| 15 | *MSH2* | NM_000251 | c.2006-6T>C | - | Intron12 | Hom | rs2303428 | Splice | Benign |
| 16 | *PMS2* | NM_000535 | c.2006+6G>A | - | Intron11 | Het | rs111905775 | Splice | Benign |
| 17 | *PMS2* | NM_000535 | c.1454C>A | p.Thr485Lys | CDS11 | Het | rs1805323 | Missense | Benign |
| 18 | *PMS2* | NM_000535 | c.1408C>T | p.Pro470Ser | CDS11 | Het | rs1805321 | Missense | Benign |
| 19 | *PMS2* | NM_000535 | c.706-4delT | - | Intron6 | Hom | rs549498051 | Splice | Benign |
| 20 | *STK11* | NM_000455 | c.920+7G>C | - | Intron7 | Het | rs2075607 | Splice | Benign |
| 21 | *TP53* | NM_000546 | c.215C>G | p.Pro72Arg | CDS3 | Het | rs1042522 | Missense | Benign |
| 22 | *BRCA1* | NM_007294 | EX8 | - | EX8 | Het | - | Deletion | Pathogenic |

*The proband aged at 58 received the 21-gene panel germline sequencing for hereditary breast or ovarian cancer syndrome at January 7^th^, 2018 after being diagnosed as ovarian cancer. The result identified a positive *BRCA1* heterozygous mutation (NM_007294) which had been reported for its pathogenicity. The germline mutation was located in the region of EX8 by autosomal recessive inheritance resulting in exon deletion and associated amino acid change. It was studied and proved in a research by Engert S et al. (Engert S, Wappenschmidt B, Betz B, Kast K, Kutsche M, Hellebrand H, Goecke TO, Kiechle M, Niederacher D, Schmutzler RK, Meindl A. MLPA screening in the BRCA1 gene from 1,506 German hereditary breast cancer cases: novel deletions, frequent involvement of exon 17, and occurrence in single early-onset cases. Hum Mutat. 2008;29(7):948-958.) The analysis in the *ATM*, *BARD1*, *BRCA1*, *BRCA2*, *BRIP1*, *CDH1*, *MSH2*, *PMS2*, *STK11* and *TP53* revealed several benign variants. The analysis in the *CHEK2,* *PALB2*, *EPCAM*, *PTEN*, *MLH1*, *MRE11A*, *MSH6*, *MUTYH*, *NBN*, *NF1*, *PMS1*, *RAD50*, *RAD51C*, *RAD51D* and *SMARCA4* revealed no variants.
